# Evaluation of Three-Dimensional Digital Models Formulated From Direct Intra-oral Scanning of Dental Arches in Comparison With Extra-oral Scanning of Poured Dental Models in Terms of Dimensional Accuracy and Reliability

**DOI:** 10.7759/cureus.54869

**Published:** 2024-02-25

**Authors:** Samer T. Jaber, Mohammad Y. Hajeer, Khaled Walid Alkhouli, Rabab Mohamad Al-Shamak, Khaldoun M.A. Darwich, Ossama Aljabban, Mohammad Khursheed Alam, Jehad M. Kara-Boulad

**Affiliations:** 1 Department of Orthodontics, Faculty of Dentistry, Al-Watanyia Private University, Hama, SYR; 2 Department of Orthodontics, Faculty of Dentistry, University of Damascus, Damascus, SYR; 3 Department of Orthodontics, Faculty of Dentistry, Syrian Private University, Damascus, SYR; 4 Department of Oral and Maxillofacial Surgery, Faculty of Dentistry, University of Damascus, Damascus, SYR; 5 Department of Preventive Dental Science, College of Dentistry, Jouf University, Sakaka, SAU; 6 Department of Orthodontics, Faculty of Dentistry, Al-Hawash Private University, Homs, SYR

**Keywords:** model analysis, precision, accuracy, reliability, digital models, orthodontic models, validation, desktop scanner, intraoral scanner, orthodontic

## Abstract

Background: The study's objective was to assess the dimensional accuracy and reliability of dental digital models prepared by direct intraoral scanning and indirect scanning of the plaster models compared to the plaster models as the gold standard.

Materials and methods: This study included 20 patients. Nine had a class I malocclusion, seven had a class II malocclusion, and four had a class III malocclusion. Intraoral scanning was done for the upper and lower arches of all the patients enrolled in this study using an intraoral scanner (i700; Medit, Seoul, Korea). The next step was preparing the plaster model for the control group. Addition-silicone impressions were taken for each patient's arches. The impressions were poured according to American Board of Orthodontics (ABO) standards. Finally, the digital models of the indirect scanning group were prepared using a 3D desktop scanner (T710; Medit). In total, 26 measurements were made on the plaster and digital models. Paired t-tests were used to test for significant differences between the studied groups. The reliability of the studied techniques was tested using intraclass correlation coefficients (ICCs). Because of the multiple comparisons, the ɑ level was adjusted and set at 0.002.

Results: No significant differences were found between the intraoral scanning group (20 patients) and the plaster models group (20 patients; P>0.002). The ICCs ranged from 0.814 to 0.993, indicating excellent agreement between the direct digital and traditional plaster models. There were no significant differences between the digital and original plaster models (P>0.002). ICCs ranged from 0.834 to 0.995, indicating excellent agreement between the indirect digital and original plaster models. No significant differences were detected between the direct and indirect digital models (P>0.002). ICCs ranged between 0.813 and 0.999, indicating excellent agreement between direct and indirect digital models.

Conclusion: Both direct and indirect scanning techniques are accurate and reliable for digital model preparation and can be considered an alternative to traditional plaster models used in clinical orthodontics diagnostic applications. The intraoral scanning technique can be considered a valid alternative for indirect scanning of the plaster models to prepare digital working models during the digital design and fabrication of orthodontic appliances such as clear aligners.

## Introduction

Orthodontists frequently use study casts for comprehensive diagnosis, treatment planning, and assessment of post-treatment results [1,2]. The use of stone casts is usually associated with some problems, including the need for adequate places for storage, vulnerability to breakage, and the possibility of loss [3,4]. However, to date, using a caliper on stone casts is considered the gold standard for diagnostic measurements in orthodontics [5]. Digitalization has become an indispensable part of the medical and dental fields in recent years, leading to the development of dental scanning techniques and digital dental casts [6]. Digital dental models can be obtained by indirectly scanning the impressions or the stone casts with the desktop lab scanners or by directly scanning the dental arches using the intraoral scanners [7,8]. Other methods of constructing 3D digital models for CBCT data have also been suggested [9]. Intraoral scanning has the advantage over the traditional plaster models and desktop scanners due to its efficiency and the ability to re-scan the missing and unclear parts of the arch without the need to make new impressions [10]. It also eliminates the impression of materials and gypsum products with easier transfer between the dental clinic and the lab [11-14]. Digital models allow clinicians to use CAD/CAM applications (computer-aided design/ computer-aided manufacturing) available for model analysis, simulation of orthodontic teeth movements, monitoring of dental movements through model superimpositions, and appliance design and fabrication, especially clear aligners [2]. Previous reviews have confirmed that the digital impressions obtained directly from intraoral scanning can be considered a viable alternative to alginate impressions for patients with completely natural dentition [15,16]. In a recent systematic review, Alassiry stated that digital impressions may not be as accurate as conventional impressions, although intraoral scanners are clinically acceptable for orthodontic treatment planning, appliances, and aligner fabrication in orthodontics [17]. These reviews recommended that more research is needed to compare digital impressions prepared using different scanners with different strategies and other conventional impression materials [16,17].

Therefore, the study's objective was to assess the dimensional accuracy and reliability of dental digital models prepared by direct intraoral scanning and indirect scanning of the plaster models compared to the plaster models as the gold standard. The null hypothesis stated that there were no significant differences between the digital models prepared by direct and indirect scanning techniques and the original plaster models regarding accuracy and reliability. 

## Materials and methods

Study design and settings

This observational cross-sectional study was conducted to validate intra- and extra-oral scanning of poured models. It was conducted at the Department of Orthodontics, Faculty of Dentistry, University of Damascus, and 3DA® (3DA, Hama, Syria) digital labs. The ethical approval was obtained from the Local Research Ethics Committee of the Faculty of Dentistry, University of Damascus (Approval no. UDDS-9962-2022PG/SRC6632). Funding was obtained from the University of Damascus (Ref no. 501100020595).

Sample size calculation

Using the Minitab™ (v. 21; Minitab, LLC, State College, PA), a sample size estimation was conducted to compare differences between the dependent groups using paired t-tests. The following assumptions were used: the smallest difference requiring detection in the mesiodistal width of a tooth was 0.25 mm, a standard deviation of 0.37 from a previous study [18], and the ɑ level and study power were set at 5% and 0.80, respectively. The analysis revealed that 20 patients were required.

Patient recruitment

Examinations of 94 patients who sought orthodontic treatment at the Department of Orthodontics, Faculty of Dentistry, University of Damascus, were conducted during February and March of 2023. Initially, 57 patients were selected according to the following inclusion criteria: (1) aged between 18-35 years, (2) permanent teeth with the exception of the third molars, (3) normal crown morphology, (4) no extracted teeth, and (5) no cleft lip or palate, or other craniofacial anomalies or syndromes. After that, 20 patients were randomly selected, and informed consent was signed after they were informed about the aim of the study. Of the 20 patients recruited in this cross-sectional study, nine had a class I malocclusion, seven had a class II malocclusion, and four had a class III malocclusion.

Impressions, plaster model construction, and scanning

The first step was preparing the digital models of the direct scanning group. Intraoral scanning was done for the upper and lower arches of all the patients enrolled in this study using a Medit intraoral scanner (i700; Medit, Seoul, Korea). The intraoral scanner was calibrated before each scan session. Scanning was continuous, starting from the occlusal surface of the lower left posterior teeth, followed by the anterior teeth with an alternating labio-lingual movement, and finally, the occlusal surface of the lower right posterior teeth. All scans were visually checked on screen, followed by re-scanning whenever a flaw was identified. The next step was preparing the plaster model for the control group. Addition silicone impressions (Zhermack, Elite HD+, Rome, Italy) were taken for each patient's arches. The impressions were poured using superhard gypsum material based on standard operation, considering that there were no broken teeth, air bubbles, or voids on the casts, and they were trimmed and polished according to American Board of Orthodontics (ABO) standards. Finally, the digital models of the indirect scanning group were prepared using a 3D desktop scanner (T710; Medit) with a precision of 7 μm (Indirect scanning group). Digital files obtained using both scanning techniques were converted into stereolithographic (.stl) format and imported to the OrthoanalyzerTM software (3Shape, Copenhagen, Denmark). All the plaster and digital models were prepared by the first author (STJ), who was sufficiently qualified in these procedures, before starting the study.

Outcome measures

**Figure 1:**
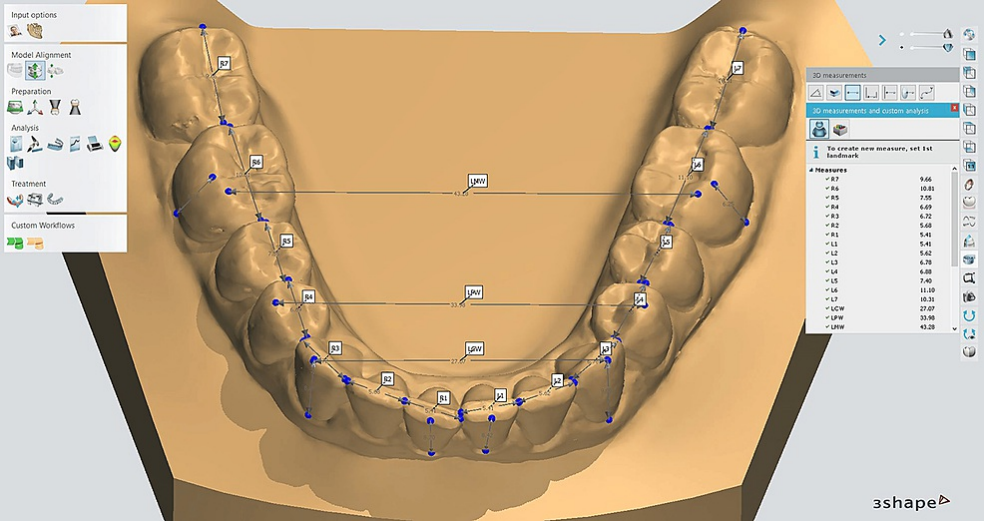
Measurements of the digital models using 3Shape Orthoanalyzer software

Measurements included in the study are summarized in Table 1. All the measurements were performed by the principal author, who was trained sufficiently in using both methods: (1) measuring the plaster models with a digital caliper (OO4OO-EEP, Orthopli, Philadelphia, PA) with an accuracy of 0.01 mm and (2) measuring the digital models using 3Shape™ Orthoanalyzer software. The computer had a 17-inch screen with 1920*1080-pixel resolution and 64-bit color (Figure 1). In-process magnification or zooming in the desired model area allowed maximal resolution. Any identification codes on the models were removed or covered, and a study identification code was given to the models by another person not involved in this research project. Investigator blinding was not possible during the study, and to combat any bias during measurement, all the plaster models were measured at once before measuring the digital models.

**Table 1:** Definitions of the linear measurements made on the plaster or digital models. Measurements in mm

Measurement	Tooth no.	Definition
Mesiodistal diameter for the upper teeth	1	Average of the right and left maximum distance between the mesial and distal points of the upper central incisors
	2	Average of the right and left maximum distance between the mesial and distal points of the upper lateral incisors
	3	Average of the right and left maximum distance between the mesial and distal points of the upper canines
	4	Average of the right and left maximum distance between the mesial and distal points of the upper first premolars
	5	Average of the right and left maximum distance between the mesial and distal points of the upper second premolars
	6	Average of the right and left maximum distance between the mesial and distal points of the upper first molars
Mesiodistal diameter for the lower teeth	1	Average of the right and left maximum distance between the mesial and distal points of the lower central incisors
	2	Average of the right and left maximum distance between the mesial and distal points of the lower lateral incisors
	3	Average of the right and left maximum distance between the mesial and distal points of the lower canines
	4	Average of the right and left maximum distance between the mesial and distal points of the lower first premolars
	5	Average of the right and left maximum distance between the mesial and distal points of the lower second premolars
	6	Average of the right and left maximum distance between the mesial and distal points of the lower first molars
Clinical crown height for the upper teeth	1	Average of the right and left maximum distance between the midpoint of the incisor margin to the lowest edge of the gingiva of the upper central incisors
	3	Average of the right and left maximum distance between the cusp tip to the lowest edge of the gingiva of the upper canines
	6	Average of the right and left maximum distance between the midpoint of the line connecting the mesial and distal buccal tips to the lowest edge of the gingiva of the upper first molars
Clinical crown height for the lower teeth	1	Average of the right and left maximum distance between the midpoint of the incisor margin to the lowest edge of the gingiva of the lower central incisors
	3	Average of the right and left maximum distance between the cusp tip to the lowest edge of the gingiva of the lower canines
	6	Average of the right and left maximum distance between the midpoint of the line connecting the mesial and distal buccal tips to the lowest edge of the gingiva of the lower first molars
Maxillary intercanine width		Distance between the right and left cusp tips of the upper canines
Maxillary interpremolar width		Distance between the right and left buccal cusp tips of the upper first premolars
Maxillary intermolar width		Distance between the right and left mesiobuccal cusp tips of the upper first molars
Mandibular intercanine width		Distance between the right and left cusp tips of the lower canines
Mandibular interpremolar width		Distance between the right and left buccal cusp tips of the lower first premolars
Mandibular intermolar width		Distance between the right and left mesiobuccal cusp tips of the lower first molars
Overjet		Distance between the incisal edge of the most anterior upper incisor to the labial surface of the corresponding lower incisor.
Overbite		Longest vertical overlap between upper and lower incisors

Statistical analysis

Data analysis was done using SPSS (v. 25.0; Armonk, NY) and Minitab™. Data distributions were tested using the Anderson-Darling test. Paired t-tests were used to test for significant differences between the studied groups and the gold-standard plaster models. The reliability of the studied techniques was tested using the intraclass correlation coefficients (ICCs). Because of the multiple comparisons, the ɑ level was adjusted and set at 0.002.

Errors of the method

Eight models were equally and randomly chosen from the studied groups, and re-measurements were done after two-week intervals in the same way using the same criteria in the first measurements. Paired t-tests were assessed to detect systematic errors. ICCs were used for the intra-rater reliability.

## Results

Direct intraoral scanning models versus plaster models

No significant differences were found between the intraoral scanning group and the plaster models group (P>0.002). The mean differences between the two groups ranged between 0.01 and 0.19 mm when evaluating the mesiodistal diameter for the upper and lower teeth. For the clinical crown height of the upper and lower central incisor, canine, and first molars, the mean differences ranged between -0.13 and -0.33 mm. The ICCs ranged from 0.814 to 0.993, indicating excellent agreement between the direct digital models and traditional plaster models (Table 2).

**Table 2:** Descriptive statistics of the measurements made on the plaster and direct digital models (n = 20) as well as the mean differences between the direct digital models and the gold standard plaster models in conjunction with the p-values of statistical testing and the intra-class correlation coefficients Ϯ Systemic error was assessed using paired t-tests. ϮϮ Random error was assessed using intraclass correlation coefficients based on absolute agreement. *Significant at P<0.002 (Bonferroni’s correction was used for multiple comparisons). The definitions of the variables are given in Table 1. SD: standard deviation, ICC: intraclass correlation coefficient

Measurement (in mm)	Tooth no.	Plaster models (n=20)		Direct digital models (n=20)		Plaster vs. Direct		
		Mean	SD	Mean	SD	Mean difference	P-value^†^	ICC^†^^†^
Mesiodistal diameter for the upper teeth	1	8.47	0.40	8.47	0.38	0.01	0.950	0.963
	2	6.61	0.72	6.76	0.59	-0.15	0.485	0.888
	3	7.83	0.56	7.81	0.56	0.01	0.950	0.894
	4	6.73	0.63	6.72	0.63	0.00	0.989	0.814
	5	6.54	0.51	6.53	0.50	0.02	0.943	0.914
	6	9.81	0.85	9.62	1.21	0.19	0.566	0.901
Mesiodistal diameter for the lower teeth	1	5.17	0.51	5.22	0.42	-0.05	0.715	0.942
	2	5.72	0.56	5.91	0.59	-0.18	0.322	0.959
	3	6.94	0.47	7.11	0.47	-0.17	0.273	0.901
	4	7.19	0.94	7.25	1.01	-0.06	0.838	0.942
	5	7.47	2.88	7.53	2.91	-0.06	0.905	0.937
	6	10.49	1.08	10.54	1.18	-0.04	0.905	0.979
Clinical crown height for the upper teeth	1	9.26	0.65	9.55	0.98	-0.29	0.288	0.903
	3	8.16	1.02	8.83	1.01	-0.21	0.511	0.929
	6	5.68	0.56	5.98	0.59	-0.29	0.122	0.901
Clinical crown height for the lower teeth	1	7.21	1.01	7.55	1.04	-0.33	0.320	0.846
	3	9.38	1.19	9.51	1.26	-0.13	0.744	0.826
	6	6.25	0.52	6.41	0.57	-0.15	0.390	0.921
Maxillary intercanine width		34.15	1.95	34.44	1.87	-0.29	0.649	0.936
Maxillary interpremolar width		36.05	2.39	36.08	2.41	-0.08	0.916	0.940
Maxillary intermolar width		47.12	3.57	47.17	3.60	-0.05	0.967	0.993
Mandibular intercanine width		26.12	2.07	26.43	1.88	-0.27	0.676	0.959
Mandibular interpremolar width		30.21	2.11	30.22	2.01	-0.01	0.980	0.841
Mandibular intermolar width		43.32	4.54	43.40	4.43	-0.08	0.954	0.958
Overjet		2.03	1.44	2.23	1.55	-0.19	0.691	0.988
Overbite		3.08	0.99	3.11	1.08	-0.03	0.926	0.964

Indirect desktop scanning models versus plaster models

There were no significant differences between the digital and original plaster models (P>0.002). When evaluating the mesiodistal diameter for the upper and lower teeth, mean differences ranged between -0.03 and -0.22 mm. The mean differences ranged between 0.01 and 0.06 mm when evaluating the clinical crown height of the studied teeth. For the intra-arch widths comparisons, the mean differences ranged between -0.19 and -0.53 mm (Table 3). ICCs ranged from 0.834 to 0.995, indicating excellent agreement between the indirect digital and original plaster models.

**Table 3:** Descriptive statistics of the measurements made on the plaster and indirect digital models (n = 20) as well as the mean differences between the indirect digital models and the gold standard plaster models in conjunction with the p-values of statistical testing and the intra-class correlation coefficients Ϯ Systemic error was assessed using paired t-tests. ϮϮ Random error was assessed using intraclass correlation coefficients based on absolute agreement. The definitions of the variables are given in Table 1. SD: standard deviation, ICC: intraclass correlation coefficient

Measurement (in mm)	Tooth no.	Plaster models (n=20)		Indirect digital models (n=20)		Plaster vs. Indirect		
		Mean	SD	Mean	SD	Mean difference	P-value^†^	ICC^†^^†^
Mesiodistal diameter for the upper teeth	1	8.47	0.40	8.51	0.46	-0.04	0.797	0.885
	2	6.61	0.72	6.74	0.59	-0.13	0.544	0.848
	3	7.83	0.56	7.86	0.57	-0.03	0.858	0.996
	4	6.73	0.63	6.82	0.67	-0.09	0.664	0.929
	5	6.54	0.51	6.73	0.56	-0.19	0.285	0.840
	6	9.81	0.85	10.04	0.90	-0.22	0.443	0.903
Mesiodistal diameter for the lower teeth	1	5.17	0.51	5.23	0.38	-0.06	0.655	0.995
	2	5.72	0.56	5.89	0.51	-0.16	0.361	0.991
	3	6.94	0.47	6.98	0.40	-0.04	0.782	0.965
	4	7.19	0.94	7.25	0.94	-0.06	0.839	0.930
	5	7.47	2.88	7.68	2.84	-0.21	0.820	0.897
	6	10.49	1.08	10.62	1.07	-0.12	0.720	0.898
Clinical crown height for the upper teeth	1	9.26	0.65	9.29	0.67	-0.03	0.889	0.931
	3	8.16	1.02	8.59	1.02	0.02	0.949	0.977
	6	5.68	0.56	5.65	0.57	0.03	0.861	0.972
Clinical crown height for the lower teeth	1	7.21	1.01	7.21	0.98	0.01	0.988	0.947
	3	9.38	1.19	9.34	1.17	0.04	0.904	0.970
	6	6.25	0.52	6.19	0.56	0.06	0.734	0.962
Maxillary intercanine width		34.15	1.95	34.48	1.80	-0.33	0.591	0.854
Maxillary interpremolar width		36.05	2.39	36.34	2.21	-0.34	0.651	0.949
Maxillary intermolar width		47.12	3.57	47.30	3.55	-0.19	0.873	0.985
Mandibular intercanine width		26.12	2.07	26.35	2.06	-0.18	0.785	0.892
Mandibular interpremolar width		30.21	2.11	30.66	2.03	-0.44	0.507	0.937
Mandibular intermolar width		43.32	4.54	43.85	4.50	-0.53	0.719	0.938
Overjet		2.03	1.44	2.13	1.49	-0.09	0.844	0.834
Overbite		3.08	0.99	3.11	1.01	-0.02	0.929	0.932

Direct intraoral scanning models versus indirect desktop scanning models

No significant differences were detected between the direct and indirect digital models (P>0.002), with the mean differences ranging between -0.02 and 0.22 mm for the mesiodistal diameter for the upper and lower teeth, -0.17 and -0.34 mm for the clinical crown height, and -0.08 and 0.45 mm for the inter-arch widths of the studied cases (Table 4). ICCs ranged between 0.813 and 0.999, indicating excellent agreement between direct and indirect digital models.

**Table 4:** Descriptive statistics of the measurements made on the Indirect and direct digital models (n = 20) as well as the mean differences between the indirect digital models and the direct digital models in conjunction with the p-values of statistical testing and the intra-class correlation coefficients Ϯ Systemic error was assessed using paired t-tests. ϮϮ Random error was assessed using intraclass correlation coefficients based on absolute agreement. *Significant at P<0.002 (Bonferroni’s correction was used for multiple comparisons). The definitions of the variables are given in Table 1. SD: standard deviation, ICC: intraclass correlation coefficient

Measurement (in mm)	Tooth no.	Indirect digital models (n=20)		Direct digital models (n=20)		Indirect vs. Direct		
		Mean	SD	Mean	SD	Mean difference	P-value^†^	ICC^†^^†^
Mesiodistal diameter for the upper teeth	1	8.51	0.46	8.47	0.38	0.04	0.747	0.955
	2	6.74	0.59	6.76	0.59	-0.02	0.914	0.879
	3	7.86	0.57	7.81	0.56	0.04	0.810	0.877
	4	6.82	0.67	6.72	0.63	0.09	0.655	0.979
	5	6.73	0.56	6.53	0.50	0.20	0.252	0.928
	6	9.84	0.90	9.62	1.21	0.22	0.236	0.813
Mesiodistal diameter for the lower teeth	1	5.23	0.38	5.22	0.42	0.01	0.940	0.999
	2	5.89	0.51	5.91	0.59	-0.02	0.886	0.993
	3	6.98	0.40	7.11	0.47	0.13	0.362	0.973
	4	7.25	0.94	7.25	1.01	-0.01	0.994	0.986
	5	7.68	2.84	7.53	2.91	0.15	0.873	0.903
	6	10.62	1.07	10.54	1.18	0.08	0.824	0.993
Clinical crown height for the upper teeth	1	9.29	0.67	9.55	0.98	-0.26	0.344	0.979
	3	8.59	1.02	8.83	1.01	-0.24	0.471	0.935
	6	5.65	0.57	5.98	0.59	-0.33	0.090	0.958
Clinical crown height for the lower teeth	1	7.21	0.98	7.55	1.04	-0.34	0.308	0.984
	3	9.34	1.17	9.51	1.26	-0.17	0.656	0.950
	6	6.19	0.56	6.41	0.57	-0.21	0.249	0.994
Maxillary intercanine width		34.48	1.80	34.44	1.87	0.04	0.940	0.961
Maxillary interpremolar width		36.34	2.21	36.08	2.41	0.25	0.733	0.963
Maxillary intermolar width		47.30	3.55	47.17	3.60	0.14	0.906	0.853
Mandibular intercanine width		26.35	2.06	26.43	1.88	-0.08	0.894	0.816
Mandibular interpremolar width		30.66	2.03	30.22	2.01	0.43	0.514	0.836
Mandibular intermolar width		43.85	4.50	43.40	4.43	0.45	0.760	0.814
Overjet		2.13	1.49	2.23	1.55	-0.10	0.840	0.971
Overbite		3.11	1.01	3.11	1.08	-0.01	0.994	0.966

The error of the method

No significant differences were found between the repeated measurements (p>0.05), indicating that systematic errors were absent and a single measurement was enough to proceed. The ICCs between the two sets of measurements ranged between 0.901 and 0.998, indicating that the interrater reliability was excellent.

## Discussion

Using an intraoral 3D digital scanner is the easiest and fastest way to obtain digital models directly from the patient without needing impression materials. However, to date, the spread of this technology is still limited in dental clinics [6]. Digital models can also be prepared by directly scanning the impressions, eliminating the need to cast and prepare plaster models. Still, transferring the occlusal relationship between the jaws to the digital models in this scanning technique is difficult [19,20]. The third technique, which is considered more prevalent, is the 3D scanning of plaster models using desktop laboratory scanners, which rely on scanning the upper and lower models separately and then scanning both of them in the regular occlusion to take an accurate replica of the dental arches and the occlusal relationship between them [21,22].

Many studies have defined or established the range of clinically acceptable measurement errors when replicating plaster models or when digital models are 3D printed for diagnostic purposes. According to the standards of the ABO for grading the plaster models, an error in intra-arch distances of no more than 0.5 mm from the gold standard (i.e., the original plaster models) is considered acceptable from a clinical perspective. Rebong et al. show a clinically significant 0.5 mm measurement difference between the studied and original models [23]. Many studies have shown that a difference ranging from 0.2 to 0.5 mm is acceptable regarding clinical accuracy [8,24]. But when it comes to the fabrication of orthodontic appliances, especially clear aligners, these differences in measurements between the digital and the original plaster models become more important because maximum transitional teeth movements with clear aligners range between 0.25-0.30 mm for each tooth in each aligner [25-27]. Therefore, the range of difference in measurements between the digital, printed, and original models should not exceed the range of 0.25-0.30 mm to ensure a correct fit of the aligners so that they exert proper orthodontic forces on the teeth according to the digitally planned predictions. According to that, when digital models are proposed to be used as working models for clear aligner fabrication, the scope of differences between the digital and the original models must not exceed 0.25 mm in the inter-dental, overjet, and overbite measurements. At the same time, a 0.5 mm dimensional discrepancy should be acceptable for the intra-arch measurements.

The study showed no significant differences between the original gypsum and the digital models prepared using the two studied scanning techniques for all measurements. No differences of clinical importance were noted when comparing the mesiodistal diameter and intra-arch widths between the three model groups. At the same time, some occlusal-gingival measurements exceeded the threshold of clinical acceptance when comparing the studied techniques, noting that the number of measurements and the amount of increase above the threshold was small. The findings of this study are consistent with the findings of Reuschl et al., Czarnota et al., and Liang et al., which showed that digital models prepared using indirect scanning of plaster models are considered accurate and reliable, allowing them to be used as an alternative to gypsum models, and the differences between plaster and digital models were not significant from a clinical point of view [28-30].

Finally, based on the results of this study, both the indirect 3D scanning techniques for plaster models and the 3D intraoral scanning techniques can be considered accurate and highly reliable. Considering the extent of the differences between these techniques when compared to each other and compared to the plaster models as the gold standard, the differences in clinical importance were very low. In addition, the differences noted between the studied techniques were similar to previous studies that used similar measurement methods, and the differences were distributed among the various measurements performed and were not concentrated within only a specific group of these measurements.

Limitations of the current study

The reproducibility of the taken impressions and the reliability of the scanning process were not evaluated in the current study, which may be considered a limitation. No reference points were used on the plaster and digital models, which might have affected the accuracy of the measurements due to identification errors. The study was limited to Medit's intraoral and desktop scanners, which might affect the generalizability of this study's results. Future studies might consider the evaluation of more scanners and their validity for clinical and diagnostic applications in the orthodontic field.

## Conclusions

Direct intraoral and indirect desktop scanning techniques are accurate and reliable for digital model preparation and can be considered an alternative to the traditional plaster models in clinical orthodontics diagnostic applications. The intraoral scanning technique can be considered a valid alternative for indirect scanning of the plaster models to prepare digital working models during the digital design and fabrication of orthodontic appliances such as clear aligners.
